# Association between radiotherapy timing and intracranial outcomes in EGFR-mutant NSCLC with brain metastases treated with EGFR-TKIs

**DOI:** 10.3389/fonc.2026.1708604

**Published:** 2026-06-22

**Authors:** Qi Liu, Jian Shi, Ruiyu Liu, Zhuofan Wang, Rong Qiu, Yunfan Wu, Juan Li, Yuxiang Wang

**Affiliations:** 1 Department of Radiation Oncology, the Fourth Hospital of Hebei Medical University & Hebei Clinical Research Center for Radiation Oncology, Shijiazhuang, China; 2 Department of Oncology, the Fourth Hospital of Hebei Medical University, Shijiazhuang, China

**Keywords:** brain metastases, delayed RT, early RT, epidermal growth factor receptor, non-small cell lung cancer

## Abstract

**Objective:**

This study evaluated the treatment outcomes and prognostic factors that influence the overall survival (OS) and intracranial progression-free survival (iPFS) in patients with epidermal growth factor receptor (*EGFR*)-mutated non-small cell lung cancer (NSCLC) and brain metastases (BMs) who were treated with radiotherapy (RT) combined with EGFR tyrosine kinase inhibitors (EGFR-TKIs).

**Methods:**

The clinical data obtained from patients diagnosed with *EGFR*-mutated NSCLC and BMs who underwent local or whole-brain RT between January 2015 and December 2020 were retrospectively reviewed. Statistical analysis was performed using SPSS version 26.0.

**Results:**

A total of 135 patients were included. Among them, 70 patients (51.9%) received early RT, while 65 patients (48.1%) received delayed RT. All patients were treated with EGFR-TKIs, either before or during RT. In the univariate analysis, the *EGFR* mutation subtype and the extent of metastases were associated with OS (*p* < 0.05), while the mutation subtype and the timing of RT were linked to iPFS. The multivariate analysis confirmed that exon 19 deletions and limited metastatic disease were independent prognostic factors for OS and that early RT independently improved iPFS (*p* < 0.05). Patients who received early RT had longer iPFS compared with patients who received delayed RT (14.6 *vs.* 10.4 months, *p* = 0.010). The median OS was also superior in patients with exon 19 deletions compared to patients with exon 21 L858R mutations (37.5 *vs.* 25.1 months, *p* = 0.009).

**Conclusion:**

The combination of EGFR-TKIs with early RT can enhance the intracranial disease control in patients with *EGFR*-mutated NSCLC and BMs. Furthermore, patients with exon 19 deletions had a more favorable prognosis compared with patients who harbored exon 21 L858R mutations.

## Introduction

Lung cancer remains the leading cause of cancer incidence and mortality worldwide (1). Non-small cell lung cancer (NSCLC) accounts for roughly 85% of all cases, with adenocarcinoma and squamous cell carcinoma being the most frequent histological types (2). Among patients with adenocarcinoma, mutations in the epidermal growth factor receptor (*EGFR*) gene are the most common, followed by rearrangements in the anaplastic lymphoma kinase (*ALK*) gene (3). In Asian populations, the prevalence of *EGFR* mutations in NSCLC has been reported to be approximately 31% (4).

Brain metastases (BMs) are a frequent complication of NSCLC, which occur in approximately 40% of patients during the disease course, with 10%–20% of patients presenting with BMs at the time of initial diagnosis (2). The incidence of BMs is even higher in patients who harbor driver gene mutations (5, 6). Without treatment, the median overall survival (OS) of NSCLC patients with BMs is only 1–2 months (7). Standard whole-brain radiotherapy (WBRT) can extend survival to approximately 7 months (8). However, in the era of targeted therapy, the treatment landscape has evolved substantially. For patients with limited BMs (typically one to four lesions), stereotactic radiosurgery (SRS) is presently recommended as the preferred local treatment due to its superior intracranial control and lower risk of neurocognitive decline when compared with WBRT (9).

EGFR tyrosine kinase inhibitors (EGFR-TKIs) have shown significant intracranial efficacy in patients with *EGFR*-mutated NSCLC and BMs (10, 11), with some studies reporting a median OS that extends up to 46 months (12). However, in the targeted therapy era, the optimal timing of radiotherapy (RT) in relation to the administration of EGFR-TKI for patients with BMs remains a matter of debate. Although the combination of EGFR-TKI and RT has been widely studied in patients with BMs, the optimal timing of RT remains unclear. The majority of prior works have focused on determining whether to add RT rather than when to initiate it relative to TKI therapy. In addition, the potential impact of timing has rarely been examined while accounting for key factors such as the metastatic burden and the *EGFR* mutation subtype. This study retrospectively compared the intracranial outcomes between patients who received early (≤1 month) and delayed (>1 month) RT in a cohort of *EGFR*-mutant NSCLC patients with BMs treated with EGFR-TKIs. Subsequently, the association between early *vs.* delayed RT and survival outcomes in patients with *EGFR*-mutated NSCLC and BMs, who were treated with combined EGFR-TKI and RT, was evaluated.

## Patients and methods

### Patient eligibility

Clinical data obtained from patients with *EGFR*-mutated NSCLC and BMs who were treated at our institution were retrospectively analyzed. This study was approved by the Ethics Committee of the Fourth Hospital of Hebei Medical University (no. 2025KT039).

The inclusion criteria were: 1) pathologically confirmed NSCLC harboring an *EGFR* mutation, with the patient receiving EGFR-TKI as systemic therapy during the study period; 2) BMs confirmed by contrast-enhanced magnetic resonance imaging (MRI) or computed tomography (CT); 3) RT for BMs performed between January 2015 and December 2020 at our institution; and 4) no prior surgical resection or RT for BMs.

The exclusion criteria were: 1) unknown or unavailable *EGFR* mutation status; 2) incomplete RT; 3) presence of secondary primary malignancies; and 4) presence of meningeal or cranial metastases or other uncontrolled serious conditions.

### Screening for leptomeningeal metastases

During data collection, patients were evaluated for leptomeningeal metastases using cranial MRI. Subjects with imaging findings suggestive of leptomeningeal involvement were excluded from the study.

### Treatment

All patients received EGFR-TKI therapy either before or during RT, including gefitinib, icotinib, or osimertinib. The RT included WBRT, local RT targeting metastatic sites, or a combination of WBRT and local RT. Three-dimensional conformal radiotherapy (3D-CRT) or intensity-modulated radiotherapy (IMRT) techniques were used.

### Definition of radiotherapy timing

Early RT was defined as treatment initiated within 1 month after BM diagnosis, while delayed RT was defined as treatment that started more than 1 month after diagnosis.

### Neuroimaging assessment

The BMs were diagnosed using contrast-enhanced MRI of the brain or, when MRI was contraindicated, contrast-enhanced CT. Intracranial treatment response was assessed according to the Response Evaluation Criteria in Solid Tumors (RECIST), version 1.1. All follow-up imaging studies were independently reviewed and measured by two independent radiation oncologists with expertise in central nervous system (CNS) malignancies. Any discrepancies were resolved by consensus or, when necessary, adjudicated by a senior neuroradiologist. Baseline imaging was obtained within 4 weeks prior to the initiation of RT. The first post-treatment assessment was performed at 1–3 months after completion of RT, with subsequent evaluations conducted every 3–6 months or as clinically indicated.

### Evaluation of therapeutic effect

The clinical efficacy of RT was assessed using the RECIST, version 1.1. Acute toxicities during RT were graded according to the acute radiation morbidity scoring criteria issued by the Radiation Therapy Oncology Group (RTOG).

### Statistical analysis

Follow-up was conducted through 31 July 2025, with a median follow-up duration of 86.2 months. OS was defined as the interval from the date of BM diagnosis to death from any cause. Patients who were alive at the last follow-up were censored. Intracranial progression-free survival (iPFS) was defined as the time from completion of cranial RT to progression of existing intracranial lesions or the development of new intracranial lesions. Patients without intracranial progression at the last follow-up were censored.

The statistical analysis was performed using SPSS software (version 26.0). Categorical variables were analyzed with the chi-square test. The survival analysis was performed using the Kaplan–Meier method for univariate analysis, while multivariate survival analysis with hazard ratios (HRs) was conducted using the Cox regression model. A two-sided *p*-value of <0.05 was considered statistically significant.

## Results

### Clinical characteristics of the patients

**Figure 1:**
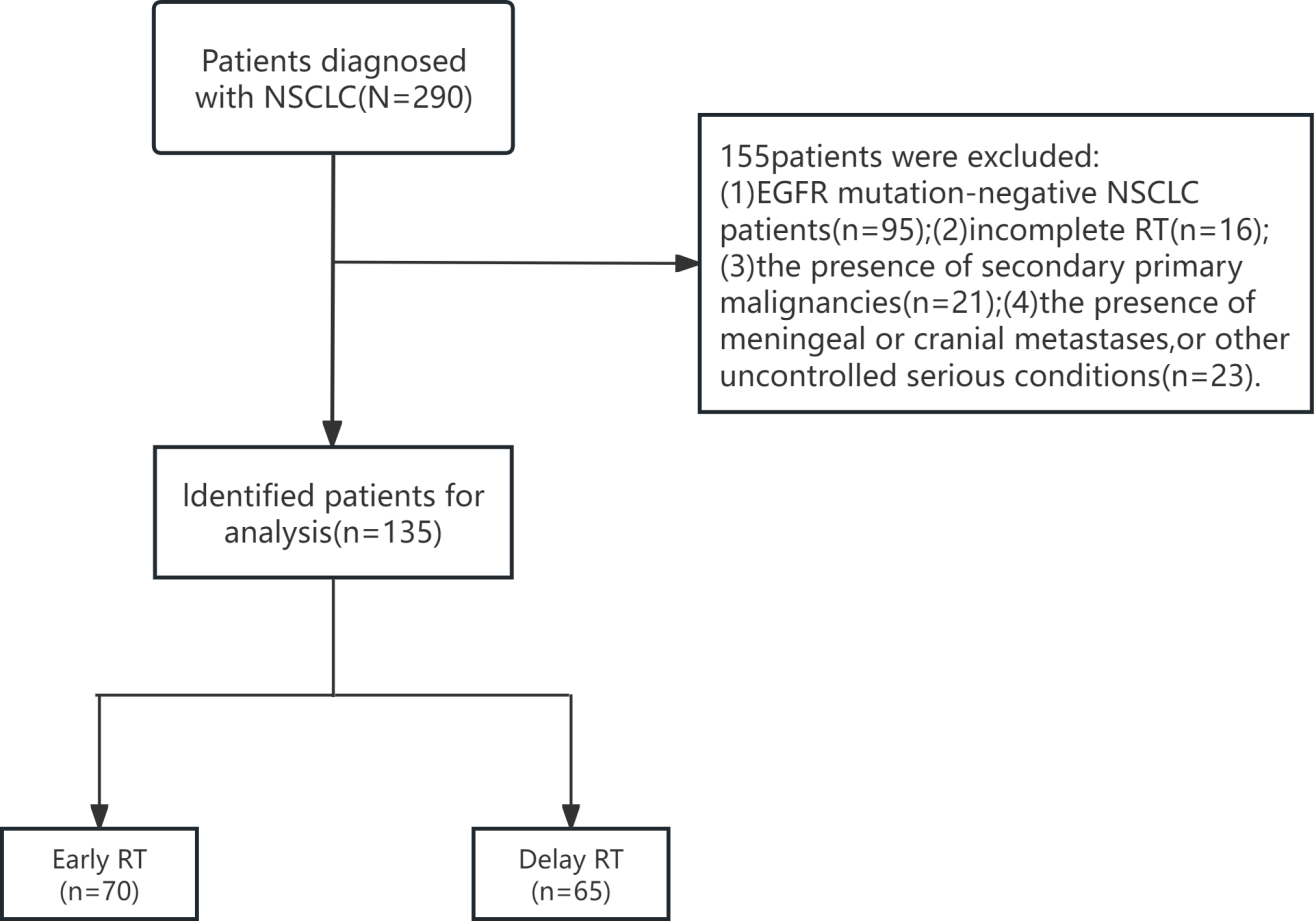
Flowchart of the experiment.

A total of 135 eligible patients were enrolled (**Figure 1**), with a median age of 61 years [range = 30–83 years, interquartile range (IQR) = 12.25]. There were 57 men and 78 women, and 45 patients had a history of smoking. The pathology revealed adenocarcinoma in 130 patients, squamous cell carcinoma in three patients, and adenosquamous carcinoma in two patients. For the treatment regimen, 38 patients received WBRT, 30 patients received local RT, and 67 patients received WBRT combined with local RT (**Table 1**). The median radiation doses were 40 Gy (range = 30–45 Gy, IQR = 5.0), 54 Gy (range = 30–60 Gy, IQR = 12.75), and 54.5 Gy (range = 42–60 Gy, IQR = 10.0), respectively. Furthermore, 115 patients (85.2%) received first-generation EGFR-TKIs, three patients (2.2%) received second-generation TKIs, and 17 patients (12.6%) received third-generation TKIs. The predominance of first-generation TKIs reflects the treatment patterns during the study period (2015–2020), i.e., before third-generation TKIs were widely adopted as the first-line therapy. At the last follow-up, nine patients were alive, while 126 patients died. The causes of death included cerebral infarction (one case), intestinal obstruction (one case), pulmonary infection (two cases), and disease progression leading to multiple organ failure (for the remaining patients).

**Table 1:** Baseline clinical characteristics of the 135 patients.

Characteristics	*n* (%)	Characteristics	*n* (%)
Gender		Neurologic symptoms	
Male	57 (42.2)	No	30 (22.2)
Female	78 (57.8)	Yes	105 (77.8)
Age (years)		Initial BMs	
≤60	66 (48.9)	No	35 (25.9)
>60	69 (51.1)	Yes	100 (74.1)
Smoking status		Systemic metastasis	
No	90 (66.7)	Oligometastasis	75 (55.6)
Yes	45 (33.3)	Polymetastasis	60 (44.4)
KPS score		Timing of RT	
≤80	72 (53.3)	Early RT	70 (51.9)
>80	63 (46.7)	Delayed RT	65 (48.1)
GPA score		RT modality	
0–1.5	91 (67.4)	WBRT	38 (28.2)
2–4	44 (32.6)	Local RT	30 (22.2)
Pathological type		WBRT + local RT	67 (49.6)
Adenocarcinoma	130 (96.3)	EGFR mutation	
Non-adenocarcinoma	5 (3.7)	19 del	53 (39.3)
No. of BMs		21 L858R	72 (53.3)
≤3	43 (31.9)	Others	10 (7.4)
>3	92 (68.1)		
EGFR-TKI generation			
First generation	115 (85.2)		
Second generation	3 (2.2)		
Third generation	17 (12.6)		

*BM*, brain metastases; *KPS*, Karnofsky Performance Status; *RT*, radiotherapy; *GPA*, graded prognostic assessment; *WBRT*, whole-brain radiotherapy; *EGFR*, epidermal growth factor receptor.

### Total response of treatment

According to the RECIST version 1.1, the intracranial metastatic lesions after RT had partial response (PR) in 61 patients (45.2%), stable disease (SD) in 69 patients (51.1%), and progressive disease (PD) in five patients (3.7%). The overall response rate (ORR) was 45.2%, and the disease control rate (DCR) was 96.3%. Furthermore, treatment-related adverse events occurred in 49 patients (36.3%, 49/135) during RT. Among these patients, 38 developed symptoms of intracranial hypertension, which were mainly dizziness, headache, nausea, and vomiting. In addition, 10 patients experienced grade 1–2 myelosuppression, while one patient experienced grade 3 myelosuppression. No patient developed symptomatic radiation necrosis, and there were no grade 4 or 5 treatment-related adverse events. Overall, the combination regimen was generally well tolerated. .

### Overall survival and intracranial progression-free survival

**Figure 2:**
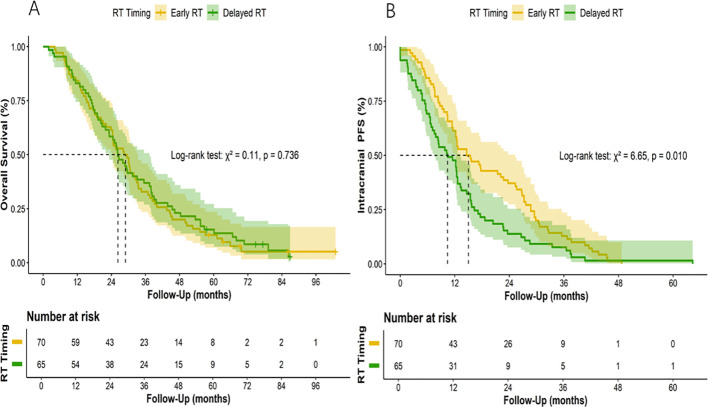
Kaplan–Meier survival curves for the entire cohort. **(A)** Overall survival (OS). **(B)** Intracranial progression-free survival (iPFS).

For the entire cohort, the 1-, 3-, and 5-year OS rates were 83.7%, 34.8%, and 14.1%, respectively, with a median OS of 28.0 months (**Figure 2**). The univariate analysis indicated that the *EGFR* mutation type and the presence of extensive metastases were associated with OS (*p* < 0.05) (**Table 2**). The multivariate analysis identified exon 19 deletion and oligometastasis as independent favorable factors for OS (*p* < 0.05) (**Table 3**).

**Table 2:** Univariate analysis of the factors associated with overall survival.

Characteristics	HR (95%CI)	*χ* ^2^	*p*
Gender			
Male	1		
Female	1.012 (0.708–1.446)	0.004	0.948
Age (years)			
≤60	1		
>60	1.213 (0.854–1.722)	1.167	0.280
Smoking status			
No	1		
Yes	0.959 (0.659–1.397)	0.047	0.829
KPS score			
≤80	1		
>80	0.840 (0.591–1.195)	0.939	0.333
GPA score			
0–1.5	1		
2–4	0.704 (0.481–1.031)	3.254	0.071
No. of BMs			
≤3	1		
>3	0.966 (0.665–1.404)	0.033	0.856
Neurologic symptoms			
No	1		
Yes	1.353 (0.871–2.102)	1.809	0.179
*EGFR* mutation		6.915	0.032
19 del	1		
21 L858R	1.602 (1.106–2.321)	6.214	0.013
Others	0.942 (0.423–2.097)	0.022	0.883
RT modality		2.007	0.367
WBRT	1		
Local RT	0.724 (0.438–1.196)	1.590	0.207
WBRT + local RT	0.978 (0.648–1.474)	0.012	0.914
Initial BMs			
No	1		
Yes	0.676 (0.452–1.009)	3.666	0.056
Systemic metastasis			
Oligometastasis	1		
Polymetastasis	1.560 (1.094–2.223)	6.031	0.014
Timing of RT			
Early RT	1		
Delayed RT	0.941 (0.663–1.337)	0.114	0.736

*HR*, hazard ratio; *95%CI*, confidence interval; *KPS*, Karnofsky Performance Status; *GPA*, graded prognostic assessment; *BM*, brain metastases; *EGFR*, epidermal growth factor receptor; *WBRT*, whole-brain radiotherapy; *RT*, radiotherapy.

**Table 3:** Multivariate analysis of the factors that independently affected overall survival.

Characteristics	HR (95%CI)	*χ* ^2^	*p*
*EGFR* mutation		6.755	0.034
19 del	1		
21 L858R	1.544 (1.063–2.244)	5.194	0.023
Others	0.785 (0.347–1.774)	0.340	0.560
Metastasis			
Oligometastasis	1		
Polymetastasis	1.582 (1.096–2.284)	6.004	0.014

*HR*, hazard ratio; 95%*CI*, confidence interval; *EGFR*, epidermal growth factor receptor.

The 1-, 3-, and 5-year iPFS rates were 54.8%, 10.4%, and 0.7%, respectively, with a median iPFS of 12.4 months (**Figure 2**). The univariate analysis indicated that the *EGFR* mutation type and the timing of RT for BMs were associated with iPFS (*p* < 0.05) (**Table 4**). The multivariate analysis revealed that early RT was an independent favorable factor for iPFS (*p* < 0.05) (**Table 5**).

**Table 4:** Univariate analysis of the factors associated with intracranial progression-free survival.

Characteristics	HR (95%CI)	*χ* ^2^	*p*
Gender			
Male	1		
Female	0.855 (0.606–1.208)	0.789	0.374
Age (years)			
≤60	1		
>60	1.052 (0.748–1.478)	0.084	0.772
Smoking status			
No	1		
Yes	1.134 (0.791–1.626)	0.470	0.493
KPS score			
≤80	1		
>80	0.763 (0.541–1.076)	2.378	0.123
GPA score			
0–1.5	1		
2–4	1.061 (0.737–1.526)	0.101	0.751
No. of BMs			
≤3	1		
>3	0.820 (0.568–1.183)	1.127	0.288
Neurologic symptoms			
No	1		
Yes	1.219 (0.801–1.856)	0.853	0.356
*EGFR* mutation		6.376	0.041
19 del	1		
21 L858R	1.102 (0.769–1.579)	0.168	0.596
Others	2.425 (1.216–4.835)	6.327	0.012
RT modality		0.331	0.847
WBRT	1		
Local RT	0.958 (0.590–1.556)	0.030	0.863
WBRT + local RT	1.079 (0.722–1.614)	0.139	0.710
Initial BMs			
No	1		
Yes	0.762 (0.514–1.129)	1.837	0.175
Systemic metastasis			
Oligometastasis	1		
Polymetastasis	1.109 (0.785–1.569)	0.345	0.557
Timing of RT			
Early RT	1		
Delayed RT	1.562 (1.107–2.203)	6.459	0.011

*HR*, hazard ratio; 95%*CI*, confidence interval; *KPS*, Karnofsky Performance Status; *GPA*, graded prognostic assessment; *BM*, brain metastases; *EGFR*, epidermal growth factor receptor; *WBRT*, whole-brain radiotherapy; *RT*, radiotherapy.

**Table 5:** Multivariate analysis of the factors that independently affected intracranial progression-free survival (iPFS).

Characteristics	HR (95%CI)	*χ* ^2^	*p*
*EGFR* mutation		4.685	0.096
19 del	1		
21 L858R	1.076 (0.743–1.559)	0.151	0.698
Others	2.165 (1.072–4.373)	4.638	0.031
Timing of RT			
Early RT	1		
Delayed RT	1.721 (1.168–2.536)	7.546	0.006

*HR*, hazard ratio; 95%*CI*, confidence interval; *EGFR*, epidermal growth factor receptor; *RT*, radiotherapy.

### Time of radiotherapy and survival

In the entire cohort, 70 patients (51.9%) received early RT, while 65 patients (48.1%) received delayed RT. Patients in the early RT group had higher proportions of initial diagnosis with BMs, neurological symptoms, and the use of WBRT with local boost (*p* < 0.05). The other baseline characteristics were similar between the two groups (*p* > 0.05) (**Table 6**).

**Table 6:** Comparison of the clinical characteristics between the early and delayed radiotherapy groups.

Characteristics	Early RT (*n* = 70), *n* (%)	Delayed RT (*n* = 65), *n* (%)	*χ* ^2^	*p*
Gender			0.024	0.877
Male	30 (42.9)	27 (41.5)		
Female	40 (57.1)	38 (58.5)		
Age (years)			2.710	0.100
≤60	39 (55.7)	27 (41.5)		
>60	31 (44.3)	38 (58.5)		
KPS score			2.239	0.135
≤80	33 (47.1)	39 (60.0)		
>80	37 (52.9)	26 (40.0)		
GPA score			0.190	0.663
0–1.5	46 (65.7)	45 (69.2)		
2–4	24 (34.3)	20 (30.8)		
No. of BMs			0.721	0.396
≤3	20 (28.6)	23 (35.4)		
>3	50 (71.4)	42 (64.6)		
Neurologic symptoms			3.563	0.059
No	11 (15.7)	19 (29.2)		
Yes	59 (84.3)	46 (70.8)		
*EGFR* mutation			1.141	0.565
19 del	30 (42.9)	23 (35.4)		
21 L858R	36 (51.4)	36 (55.4)		
Others	4 (5.7)	6 (9.2)		
RT modality			11.704	0.003
WBRT	12 (17.1)	26 (40.0)		
Local RT	14 (20.0)	16 (24.6)		
WBRT + local RT	44 (62.9)	23 (35.4)		
Initial BMs			26.709	<0.001
No	5 (7.1)	30 (46.2)		
Yes	65 (92.9)	35 (53.8)		
Systemic metastasis			0.095	0.758
Oligometastasis	38 (54.3)	37 (56.9)		
Polymetastasis	32 (45.7)	28 (43.1)		

*RT*, radiotherapy; *KPS*, Karnofsky Performance Status; *BM*, brain metastases; *EGFR*, epidermal growth factor receptor; *WBRT*, whole-brain radiotherapy.

**Figure 3:**
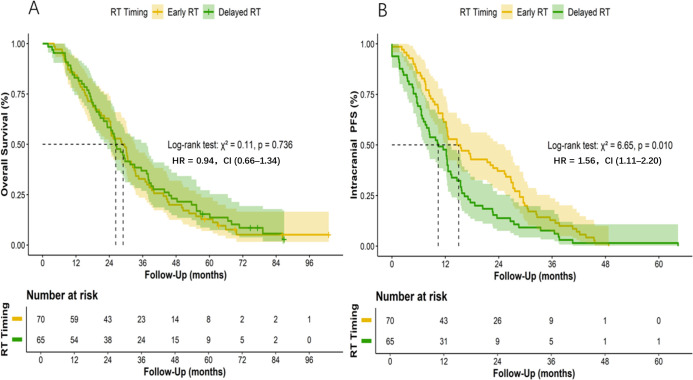
Kaplan–Meier survival curves for the comparison of the early and delayed radiotherapy groups. **(A)** Overall survival (OS). **(B)** Intracranial progression-free survival (iPFS).

The median OS and the 1-, 3-, and 5-year OS rates were 28.4 months and 84.3%, 32.9%, and 12.9%, respectively, in the early RT group, while these were 26.3 months and 83.1%, 36.9%, and 15.4%, respectively, in the delayed RT group (*p* = 0.736). The median iPFS and the 1-, 3-, and 5-year iPFS rates were 14.6 months and 61.4%, 12.9%, and 0%, respectively, in the early RT group and were 10.4 months and 47.7%, 7.7%, and 1.5%, respectively, in the delayed RT group (*p* = 0.010) (**Figure 3**). Furthermore, the intracranial objective response rate (iORR) was higher in the early RT group compared with the delayed RT group (54.3% *vs.* 35.4%, *p* = 0.027).

### Subgroup analysis

Subgroup analyses were performed according to the *EGFR* mutation subtype and the metastatic burden. Among those with exon 19 deletions, 30 patients received early RT and 23 received delayed RT. The median OS was 32.9 *vs.* 38.3 months (*p* = 0.775), while the median PFS was 21.4 *vs.* 12.4 months (*p* = 0.071). Among those with exon 21 L858R mutations, 36 patients received early RT and 36 received delayed RT. The median OS was 24.6 *vs.* 25.1 months (*p* = 0.731), while the median PFS was 12.5 *vs.* 8.4 months (*p* = 0.066). In the oligometastatic subgroup, 38 patients received early RT and 37 patients received delayed RT. The median OS was 30.6 *vs.* 29.3 months (*p* = 0.971), while the median PFS was 17.6 *vs.* 7.8 months (*p* = 0.018). Among patients with extensive metastases, 32 received early RT and 28 received delayed RT. The median OS was 21.1 *vs.* 23.3 months (*p* = 0.725), while the median PFS was 12.5 *vs.* 12.2 months (*p* = 0.240).

### Efficacy of 19-del and 21-L858R

**Figure 4:**
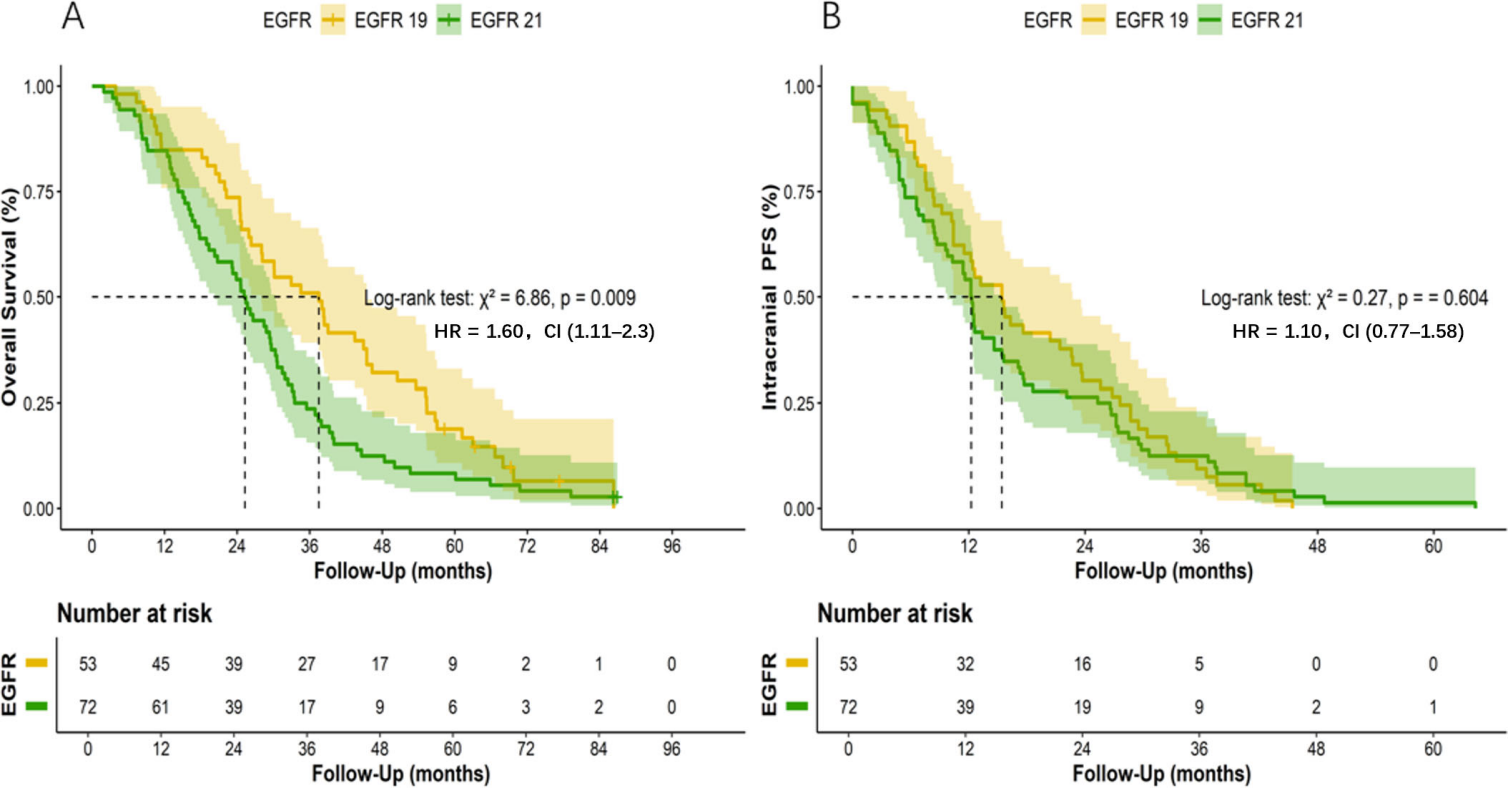
Kaplan–Meier survival curves for the comparison of the mutation subtypes. **(A)** Overall survival (OS) for patients with exon 19 deletions (*EGFR19*) *vs.* exon 21 L858R mutations (*EGFR21*). **(B)** Intracranial progression-free survival (iPFS) for patients with exon 19 deletions *vs.* exon 21 L858R mutations.

Among the patients, 53 (39.3%) had exon 19 deletions, 72 (53.3%) had the exon 21 L858R mutation, and 10 (7.4%) had other mutation types. The median OS was 37.5 months for patients with exon 19 deletions and was 25.1 months for patients with exon 21 mutations (*p* = 0.009). The median iPFS was 15.4 months for those with exon 19 deletions and was 12.2 months for those with exon 21 mutations (*p* = 0.604) (**Figure 4**).

## Discussion

Previous studies have revealed that, for NSCLC patients with *EGFR* or *ALK* mutations and BMs, combining targeted therapy with local RT provides better outcomes compared with TKIs alone. A large retrospective study of *EGFR*-mutant NSCLC patients with BMs (*n* = 1,553) reported that EGFR-TKIs combined with RT significantly prolonged the OS and iPFS compared with EGFR-TKIs alone (*p* < 0.001) (13). Similarly, Pan et al. (14) reviewed 142 *EGFR*-mutated NSCLC patients with BMs and reported median iPFS of 30.0, 14.0, and 12.0 months in the stereotactic radiotherapy (SRT)+TKIs, WBRT+TKIs, and TKIs-alone groups, respectively (*p* < 0.05). Furthermore, the SRT group had superior OS. These findings are consistent with the higher biologically effective dose of SRT, its better tumor control, and its lower risk of cognitive impairment compared with WBRT. In a prospective study, Duan et al. (15) also confirmed that concurrent intracranial RT with targeted therapy was well tolerated, with no significant effects on quality of life or cognition.

The optimal timing of RT in *EGFR*-mutated NSCLC with BMs remains a matter of debate. In this study, early RT was associated with longer iPFS (14.6 *vs.* 10.4 months, *p* = 0.010) and a higher iORR (54.3% *vs.* 35.4%, *p* = 0.027), while no significant difference in OS was observed. From a safety perspective, the combined treatment was generally well tolerated, with a low incidence of severe hematologic toxicity and no cases of symptomatic radiation necrosis. The absence of radiation necrosis may be related to the predominant use of conventionally fractionated RT techniques (3D-CRT/IMRT), which differ in toxicity profile from single-fraction SRS.

Yang et al. (16) similarly reported an improved iPFS with early RT, but without OS benefit, while Qian et al. (17) reported that early RT combined with TKIs improved both the iPFS (26.9 *vs.* 20.2 months, *p* = 0.020) and OS (31.2 *vs.* 22.3 months, *p* = 0.031). The present findings are broadly consistent with the findings of Yang et al., who reported improved iPFS without a corresponding OS benefit, and partly align with the findings of Qian et al., who observed improvements in both iPFS and OS. In contrast, Magnuson et al. reported a median OS that exceeded 40 months with early SRS combined with TKIs. This difference likely reflects the variations in patient selection, as their study included only TKI-naive patients, and the differences in RT techniques. In the present cohort, the treatment was limited to conventional fractionated RT (3D-CRT/IMRT), without the use of SRS, which may have contributed to the more modest survival outcomes observed. Moreover, in the present cohort, the progression of extracranial disease was the leading cause of death. Nearly 90% of patients had extracranial metastases, and 45.2% of the patients in the early RT group had high extracranial tumor burden, likely explaining the lack of OS improvement despite the longer iPFS. In addition to the high extracranial disease burden, several factors may account for the lack of an OS benefit despite the improved iPFS. First, the majority of patients received first-generation TKIs (85.2%), which have limited systemic efficacy when compared with newer agents. Thus, systemic progression remained common regardless of the intracranial control. Second, non-cancer-related mortality was low (only four deaths), indicating that the outcomes were largely driven by disease progression. Taken together, improved intracranial control alone may be insufficient to translate into an OS benefit when extracranial disease is not adequately controlled. Third, the post-progression treatments (e.g., chemotherapy or later-line TKIs) were not standardized and were incompletely captured, which may have influenced the OS comparisons between groups.

The prognostic role of metastatic burden has been well recognized. The 2019 EORTC consensus defined oligometastatic NSCLC as five or fewer lesions in no more than three organs (18). In this study, patients with oligometastatic disease had significantly longer OS compared to patients with polymetastatic disease (30.5 *vs.* 23.3 months, *p* = 0.013). These patients (with lower tumor burden) are more likely to benefit from the combined systemic and local treatments. This aligns with the study conducted by Sun et al. (19), which reported that the combination of thoracic RT with EGFR-TKIs in *EGFR*-mutated oligometastatic NSCLC can improve the PFS (17.1 *vs.* 10.6 months, *p* = 0.004) and OS (34.4 *vs.* 26.2 months, *p* = 0.029). A meta-analysis (20) of 115 studies also confirmed the longer survival for oligometastatic disease compared with polymetastatic disease (34.4 *vs.* 21.3 months), with the poorest outcomes observed in patients with liver or bone metastases (10.5 and 12.4 months, respectively).

For the RT techniques, the investigators observed no differences in the OS (24.6 *vs.* 30.1 *vs.* 26.2 months, *p* = 0.364) or iPFS (12.4 *vs.* 12.6 *vs.* 12.2 months, *p* = 0.845) among WBRT, local RT, and WBRT plus local boost. For patients with three or fewer BMs, WBRT was not used, and merely 7.6% of patients with more than three BMs received local RT. Ni et al. (21) reported that WBRT combined with local RT can achieve longer OS (22.2 months) compared with WBRT alone (13.7 months, *p* < 0.001) or with SRS alone (17.3 months, *p* = 0.011), as well as better iPFS. A meta-analysis (22) of 2,728 patients also revealed that WBRT plus SRS prolonged survival compared with treatment alone, although WBRT carries a risk of long-term cognitive toxicity (23). With the increasing use of SRS, this has presently become the preferred treatment for patients with one to four BMs (24). Recent studies (25, 26) have extended its use to patients with multiple lesions, showing longer survival and better quality of life when compared with WBRT. A multicenter study (27) reported that *EGFR*/*ALK*-positive NSCLC patients with four or more BMs who were treated with SRS had longer OS and fewer neurological complications and often avoided WBRT. For patients with intracranial relapse after SRS, WBRT remains a salvage option: one study (28) reported a longer OS with salvage WBRT after multiple SRS sessions compared with repeat SRS alone (17.3 *vs.* 13.8 months).

In the present cohort of *EGFR*-mutant NSCLC with BMs treated with EGFR-TKIs and RT, earlier RT was associated with longer PFS in patients with oligometastatic disease (17.6 *vs.* 7.8 months, *p* = 0.018). This pattern may reflect more effective local control when RT is delivered at a lower intracranial tumor burden, thereby limiting early intracranial progression during TKI therapy. In contrast, delayed RT may allow residual lesions to progress before local treatment is initiated.

Among patients with more extensive metastatic disease, no clear PFS advantage was observed (12.5 *vs.* 12.2 months), and OS was similar between groups (21.1 *vs.* 23.3 months). In this setting, the outcomes were likely driven largely by systemic disease, which may reduce the relative impact of local RT. These findings suggest that the potential benefit of earlier RT may be more apparent in patients with limited intracranial disease, while in those with extensive disease, systemic treatment remains as the primary determinant of outcome.

The investigators also found that patients with exon 19 deletions had better OS compared to patients with the exon 21 L858R mutation, which is consistent with previous findings. The analysis results from the LUX-Lung 3 and LUX-Lung 6 trials (29) revealed that afatinib significantly improves OS in patients with exon 19 deletions, but not in patients with L858R. Similarly, Fan et al. (30) reported a longer OS for 19-del when compared with L858R (32.7 *vs.* 27.4 months, *p* = 0.037) in *EGFR*-mutated NSCLC with BMs treated with icotinib, with or without RT.

This study has several limitations. First, its retrospective design and relatively small sample size make selection bias unavoidable. Second, due to the retrospective nature of the analysis, data on subsequent lines of systemic therapy following progression on first-line EGFR-TKIs, such as chemotherapy, immunotherapy, or enrollment in clinical trials, were not systematically collected. These post-progression treatments may have influenced the OS and could confound the interpretation of the OS outcomes, particularly the absence of a significant OS difference between the early and delayed RT groups. Third, the quality of life and neurocognitive outcomes were not systematically assessed. In addition, there was no uniform standard with regard to the choice of EGFR-TKIs or RT techniques, both of which may have affected the treatment outcomes. Finally, the local RT in this cohort was limited to conventionally fractionated techniques (3D-CRT or IMRT), without the use of SRS.

One methodological issue in the comparison of early and delayed RT is the potential for immortal time bias. Patients in the delayed group, by definition, had to survive beyond the 1-month cutoff, while patients in the early group received treatment within that period. Events that occurred during the first month could thereby affect the group assignment. In order to address this, iPFS was measured from the completion of cranial RT in both groups rather than from diagnosis. For OS, the follow-up began at the time of diagnosis, and all patients were included regardless of the RT timing. Despite these steps, some degree of bias cannot be excluded given the retrospective design.

In conclusion, the present retrospective analysis suggests that, in patients with *EGFR*-mutated NSCLC and BMs, earlier initiation of RT in combination with EGFR-TKIs is associated with improved intracranial disease control when compared with a delayed RT approach. However, this association did not translate into a measurable OS benefit in the present cohort, potentially reflecting the impact of extracranial disease burden. In addition, patients harboring exon 19 deletions appeared to have a more favorable prognosis compared to patients with exon 21 L858R mutations. Future prospective studies should address several priorities. First, randomized trials that compare upfront RT with RT deferred until intracranial progression are needed in patients receiving third-generation TKIs, given the improved CNS penetration. Second, more consistent definitions of RT timings would facilitate comparisons across studies. Finally, patient-reported outcomes and neurocognitive function should be incorporated as co-primary endpoints to better balance intracranial efficacy with potential toxicity.

## Data Availability

The raw data supporting the conclusions of this article will be made available by the authors, without undue reservation.
